# Conservative management of double teeth in molar teeth with pulp or periapical disease: a report of five cases and literature review

**DOI:** 10.1186/s12903-023-03463-4

**Published:** 2023-10-10

**Authors:** Mengxi Chen, Xinna Bai, Xiangzhu Wang, Xiaoli Xie, Minmin Chen

**Affiliations:** 1 Xiangya Stomatological Hospital, Central South University, Changsha, Hunan 410008 China; 2 Xiangya School of Stomatology, Central South University, Changsha, Hunan 410008 China; 3 Hunan Key Laboratory of Oral Health Research, Changsha, Hunan 410008 China

**Keywords:** Cone-beam computed tomography scans, Geminated teeth, Fused teeth, Double teeth, Root canal therapy, Case report

## Abstract

**Background:**

Double teeth are usually the result of an abnormality in the developing tooth germ. Double teeth can occur in either the primary or permanent dentition, with the majority of cases concerning permanent teeth reported in the anterior teeth and less frequently in the molar teeth.

**Case presentation:**

This report illustrates five cases of double teeth in molars with pulp and periapical disease, including one case of geminated teeth and four cases of fused teeth. Radiographic findings revealed the presence of extra teeth on the buccal aspect of the molar in five cases, with or without communication between the two root canal systems. Root canal treatment was performed by using CBCT and a dental operating microscope. The treatment outcome was good in all five cases.

**Conclusion:**

The diagnosis and treatment of double teeth requires special attention. The root canal system should be carefully explored to obtain a full understanding of the anatomy, allowing it to be fully cleaned and obturated. Proper anatomical structure analysis prior to treatment facilitates the development of an appropriate treatment plan, thereby increasing the likelihood of successful treatment both aesthetically and functionally.

## Background

Double teeth are usually the result of an abnormality in the developing tooth germ. The most common double teeth are geminated teeth, fused teeth, and concrescence of teeth [1]. Geminated teeth are formed by an inward depression that separates a tooth germ incompletely, manifesting as a completely or incompletely separated crown with a common root and root canal system. The incidence of gemination is approximately 0.3–0.5% in permanent dentition and 0.5–2.5% in primary dentition [2]. Geminated teeth occur mainly in the anterior teeth region and are more common in the incisal teeth region [3]. Fused teeth are often formed by the fusion of two normal tooth germs and occur during tooth development [4]. The etiology of tooth fusion is still unclear. It is now mostly thought to be associated with local pressure, trauma, and genetic predisposition [5, 6]. Fused teeth can occur in either the primary or permanent dentition, and fusion can also occur between normal teeth and supernumerary teeth, most commonly in the mandibular primary incisors and very rarely in the permanent molars. The incidence of fusion is approximately 0.1% in permanent dentition and 0.5% in primary dentition. The majority of cases of double teeth concerning permanent teeth were reported as anterior and less frequently molar. Five cases of molar double teeth with pulp or periapical disease were included in this report, with good treatment results.

## Case presentation

### Case 1

**Fig. 1:**
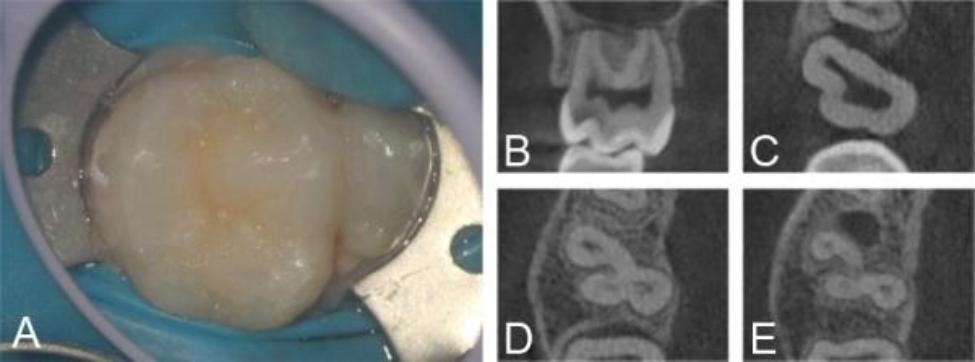
Preoperative intraoral photographs and radiographs of the geminated tooth in case 1. A: On occlusal intraoral view, an abnormal extra cusp is clearly visible on the buccal side of the crown, distinct from the crown of the right maxillary first molar by a sulcus. B: CBCT coronal cross-sections reveal a radiolucency that has already extended toward the distopulpal horn. C-E: CBCT axial cross-sections confirm that the diseased tooth shares a common root and root canal system with the supernumerary tooth

A 21-year-old female complained of a spontaneous, intermittent throbbing pain in the maxillary right molar area with worsening pain when drinking cold water that had been present for about 7 days. The medical history revealed nothing relevant. Intraoral examination revealed an abnormal extra cusp on the buccal side of the crown of the right maxillary first molar (Fig. 1A). Tooth #16 was tender on percussion, sensitive to cold tests. No mobility or swelling was evident.

CBCT imaging was performed to assess the root canal systems of the diseased tooth. The axial, sagittal, and coronal sections confirmed that the diseased tooth shared a common root and root canal system with the supernumerary tooth. Radiolucent area was not seen around the apical region. A wide pulpal image was visible in the coronal plane, and a radiolucency has already approached the distopulpal horn which may be caused by caries (Fig. 1B). The axial section showed that the pulp chamber of the supernumerary tooth was connected to tooth #16 in the cervical 1/3 plane of the root; in the middle 1/3 plane the 4 root canals were not connected to each other; in the apical 1/3 plane the root was divided into proximal root and distal root (Fig. 1C-E). Based on the clinical symptoms of spontaneous pain, sensitivity to cold tests, and imaging manifestations showing an incompletely separated crown with common roots and root canals for the diseased tooth, as well as the absence of radiolucent areas around the apical region, the tooth was diagnosed as acute pulpitis combined with geminated maxillary first molars.

### Case 2

**Fig. 2:**
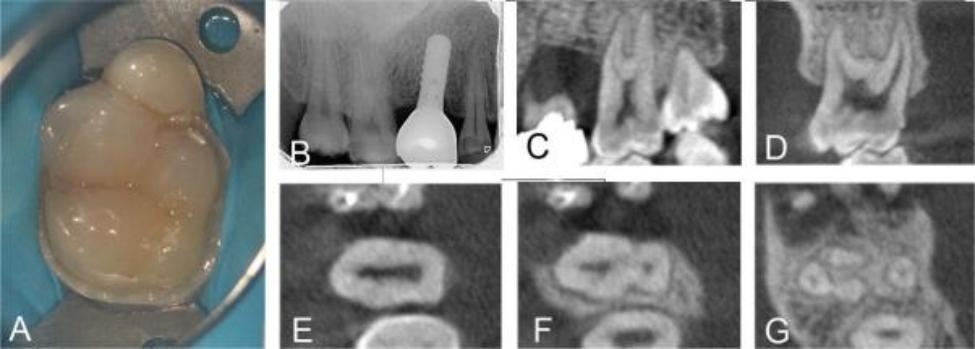
Preoperative intraoral photographs and radiographs of the fused tooth in case 2. A: In the occlusal intraoral view, a distinct developmental groove can be seen between the supernumerary tooth and the normal tooth, with no visible separation between the two; B: Through the preoperative radiograph, high-density overlapping images can be seen in the right maxillary second molar, which was suspected to be a fused tooth; C-D: CBCT sagittal and coronal cross-sections showed that diseased tooth shared the common root and root canal system with the supernumerary tooth; E-G: CBCT axial cross-sections of the fused tooth. In the apical 1/3 plane the root canal system is divided into four root canals

A 37-year-old male reported to our Oral Medicine Department with a chief complaint of pain in the maxillary right molar region for the past 2 days. The medical history revealed nothing relevant. The patient reported a history of spontaneous pain, increased pain at night, temperature sensitivity, and pain relieved after taking painkillers. Clinical examination revealed an anomalous second molar, possibly representing a fusion between the maxillary right second molar and a supernumerary tooth in the mesiobuccal aspect. Furthermore, cracks were observed on the mesial occlusal surface of the diseased tooth’s crown (Fig. 2A). Distinct developmental grooves between the supernumerary tooth and its normal counterpart were noticed. Despite the presence of these grooves, there was no discernible separation between the two. Tooth #17 was tender on percussion, sensitive to cold tests. No mobility or swelling was evident. Digital periapical radiography was performed preoperatively, and the results showed no specific pathosis. High-density overlapping images can be seen in the maxillary right second molar, in which images resembling the root canal system can be seen (Fig. 2B). Teeth fusion was suspected. The patient had an implant restoration of the right maxillary first molar in 2020, resulting in heavy artifacts in the CBCT images taken now. To obtain clear images of the 17 root canals, referring to the CBCT imaging results from 2016 showed that in the sagittal and coronal planes the diseased tooth shared the common root and root canal system with the supernumerary tooth (Fig. 2C and D). In the cervical 1/3 plane the pulp chamber of the supernumerary tooth was connected to tooth #17; in the middle 1/3 plane the root was divided into 2 root canals, which are not connected to each other; in the apical 1/3 plane the root was divided into proximal mesial, distal mesial and palatal roots (Fig. 2E-G). Based on the clinical symptoms of spontaneous pain, increased pain at night, temperature sensitivity, and imaging manifestations showing that the root canal system of supernumerary tooth and diseased tooth was completely fused above the middle 1/3 plane of the root, and separated below the apical 1/3 plane of the root, no radiolucent area around the in the apical region, the diagnosis of acute pulpitis of the maxillary fused second molars and the supernumerary tooth was made.

### Case 3

**Fig. 3:**
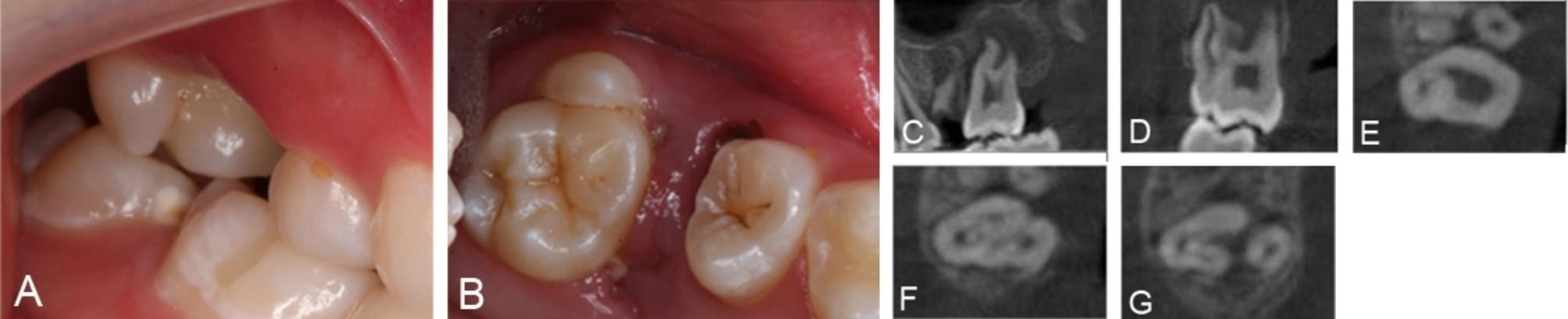
Preoperative intraoral photographs and radiographs of the fusion tooth in case 3. A,B: Buccal and occlusal intraoral view. The occlusal surface shows four intact cusps, in addition to one relatively independent cusp close to the buccal side of the crown, and the crown is free of caries, cracks or dental defects; C-D: CBCT sagittal and coronal cross-sections of the fused tooth, showing that the root canal system of the second molar and the supernumerary tooth were not connected to each other; E-G: CBCT axial cross-sections of the fused tooth.The root canal systems of the affected tooth and the multiple teeth remain independent from the crown to the root apex

A 14-year-old female patient presented to our clinic with a complaint of pain in her right maxillary molar for 1 month. She was referred to our clinic because of complex root canal morphology after having been seen in a dental clinic. The medical history revealed nothing relevant. Oral examination revealed the fusion between the buccal aspect of tooth #17 and the supernumerary tooth resulted in a wide distinct crown and grooves on the complex (Fig. 3A and B). Tooth #17 was tender on percussion. A pulp electric viability test yielded negative responses. No mobility or swelling was evident. Radiolucent area was seen around the apical region (Fig. 3C). CBCT images showed that in the coronal plane the roots of the diseased tooth and the supernumerary tooth are completely fused, but the root canal systems were independent of each other (Fig. 3D). The axial section showed two separate pulp cavity sections in the cervical 1/3 plane of the root (Fig. 3E). The supernumerary tooth had a separate pulp cavity system without connection to the pulp cavity of the second molar. In the middle 1/3 plane the root divided into buccal and palatal roots, the buccal root was “C” shaped, four root canal sections were visible without interconnection; a flat circular root canal section was visible in the palatal root (Fig. 3F and G). The root canal systems of the second molar and the supernumerary tooth were completely independent of each other. Based on the clinical manifestations of tender on percussion, negative pulp electric viability test results, and imaging manifestations showing that the root canal system of supernumerary tooth and diseased tooth was always independent, and radiolucent area was seen around the apical region, the affected tooth was diagnosed as chronic apical periodontitis of the fused tooth.The infection of the diseased tooth was considered to be a retrograde infection from the residual root of tooth #16.

### Case 4

**Fig. 4:**
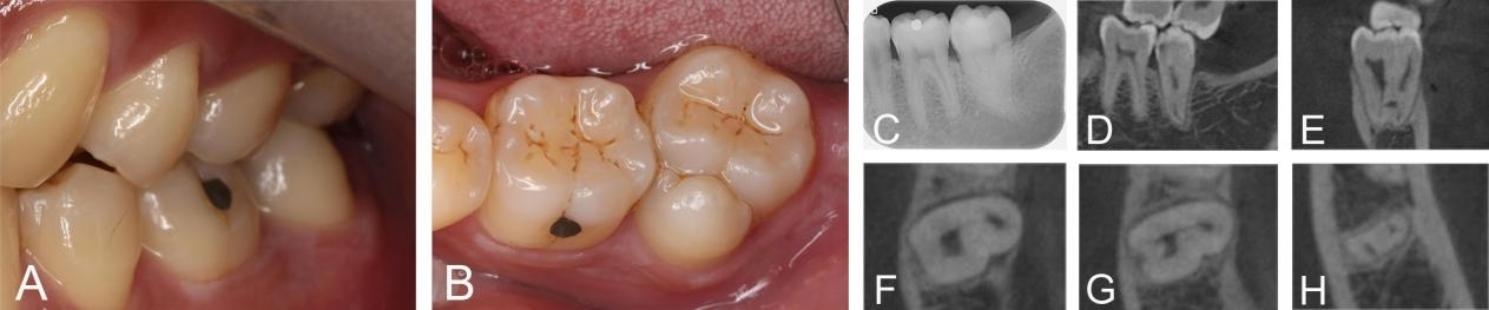
Preoperative intraoral photographs and radiographs of the fusion tooth in case 4. A,B: Buccal and occlusal intraoral view. An extra teeth on the buccal aspect of the mandibular left second molar, and them were separated by developmental grooves. C: A preoperative radiograph of Through the preoperative radiograph, high-density overlapping images can be seen in the left mandibular second molar, which was suspected to be a fused tooth; D-E: CBCT sagittal and coronal cross-sections of the fused tooth. A radiolucency was visible on the mesial surface of the diseased tooth. In the apical 1/3 plane, the two root canals are connected by transverse latera branches.; F-H: CBCT axial cross-sections of the fused tooth, showing two root canals connected by the isthmus

A 26-year-old male reported to our Oral Medicine Department with a chief complaint of pain in the mandibular left molar region for the past 4 days. The patient reported a history of spontaneous pain, increased pain at night, temperature sensitivity, and no biting pain. Clinical analyses revealed the presence of an extra teeth on the buccal aspect of the mandibular left second molar; however, they were separated by developmental grooves, but there was no clear separation between the two (Fig. 4A and B). Tooth #37 was tender on percussion, sensitive to cold tests. No mobility or swelling was evident. A preoperative periapical radiograph revealed no caries or apical pathology, but PDL widening was observed (Fig. 4C). CBCT images showed that the diseased tooth shared a common irregularly shaped flat tooth root with the supernumerary tooth. A radiolucency could be seen on the mesial surface of the diseased tooth in the sagittal plane (Fig. 4D). Coronal section showed two root canals connected in the apical 1/3 plane by a transverse lateral branch (Fig. 4E). Axial section showed two root canals connected by the isthmus, and two apical foramens were visible in the apical 1/3 plane (Fig. 4F-H). Based on the clinical symptoms of spontaneous pain, increased pain at night, temperature sensitivity, and imaging manifestations showing that the root canal system of supernumerary tooth and diseased tooth was connected by transverse latera branches in the apical 1/3 plane of the root, and a widening of the PDL was observed, the diagnosis of acute pulpitis of the mandibular fused second molars and the supernumerary tooth was made.

### Case 5

**Fig. 5:**
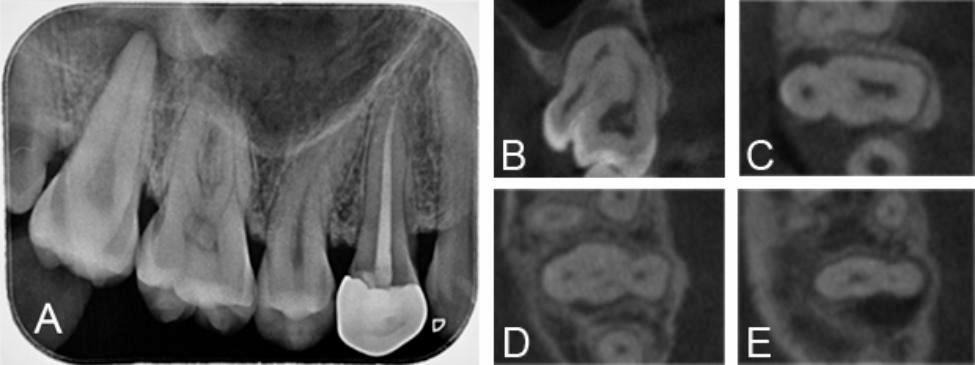
Preoperative intraoral photographs and radiographs of the fusion tooth in case 5. A: A preoperative radiograph of the right maxillary second molar. The root canal image was overlapping and blurred, and did not provide enough information; B: CBCT coronal cross-sections of the fused tooth. Two root canals did not communicate with each other, and pulp stone could be seen in the pulp chamber; C-E: CBCT axial cross-sections of the fused tooth, showing two pulpal cavities were independent of each other

A 35-year-old female reported to our Oral Medicine Department with a chief complaint of pain in the right maxillary molar region for the past 15 days. She was referred to our clinic because of complex root canal morphology after having been seen in a dental clinic. The medical history revealed nothing relevant. Clinical examination revealed an extra cusp on the buccal aspect of the maxillary right second molar. Tooth #17 was tender on percussion. A pulp electric viability test yielded negative responses. No mobility or swelling was evident. Digital periapical radiography was performed preoperatively, and the results did not provide sufficient information (Fig. 5A). CBCT images showed that the diseased tooth shared a common irregularly shaped flat tooth roots with the supernumerary tooth. Radiolucent area was seen around the apical region. Coronal section showed that the two root canals did not communicate with each other, and pulp stone could be seen in the pulp chamber of the 17# tooth (Fig. 5B). The axial section showed two pulpal cavities were independent of each other in the cervical 1/3 of the root plane (Fig. 5C); in the middle 1/3 plane the root was divided into 4 root canals (Fig. 5D); a large periapical radiolucency encompassing the distal regions could be seen (Fig. 5E). Based on the clinical symptoms of tender on percussion, negative pulp electric viability test results, and imaging manifestations showing that the root canal system of supernumerary tooth and diseased tooth did not communicate with each other, and a large periapical radiolucency encompassing the distal regions could be seen, the diagnosis of chronic apical periodontitis of the maxillary fused second molars and the supernumerary tooth was made.

The clinical conditions were explained to the five patients, and root canal therapy was proposed and conducted with the help of a dental operating microscope. The specific treatment steps were as follows. A rubber dam was properly positioned, and the entire procedure was performed under a dental microscope (ZUMAX, Suzhou, China) and with the guidance of CBCT. High-speed handpiece assisted in opening the pulp chamber, and the access opening was prepared. The working length was determined by both radiographs and an electronic apex locator (E-PEX; Eighteeth, China). The coronal access was obtained using #15/08 (Orodeka, PLEX, Italy), then the #08 and #10 K-files (Densply, United States) were used to explore and dredge the position of the canals. Next, the root canals were shaped and enlarged using a Ni-Ti file rotary system (Orodeka, PLEX, Italy) in the order of #15/03, #20/04, and #25/04, and the canals were irrigated with 0.5% NaOCl (BEKENDENT, China) during the whole procedure. Then, 5.25% NaOCl (BEKENDENT, China), 17% EDTA (BEKENDENT, China), 0.5% NaOCl, sterile water (BEKENDENT, China) and ultrasonication (P5 Newtron XS, SATELEC, France) were used for final irrigation, and the canals were dried with paper points. Then, a Ca(OH)_2_ paste (BIOTECHNOLOGY LONGLY, China) was applied to the canals, and the access cavity was sealed using GC Fuji VII.

**Fig. 6:**
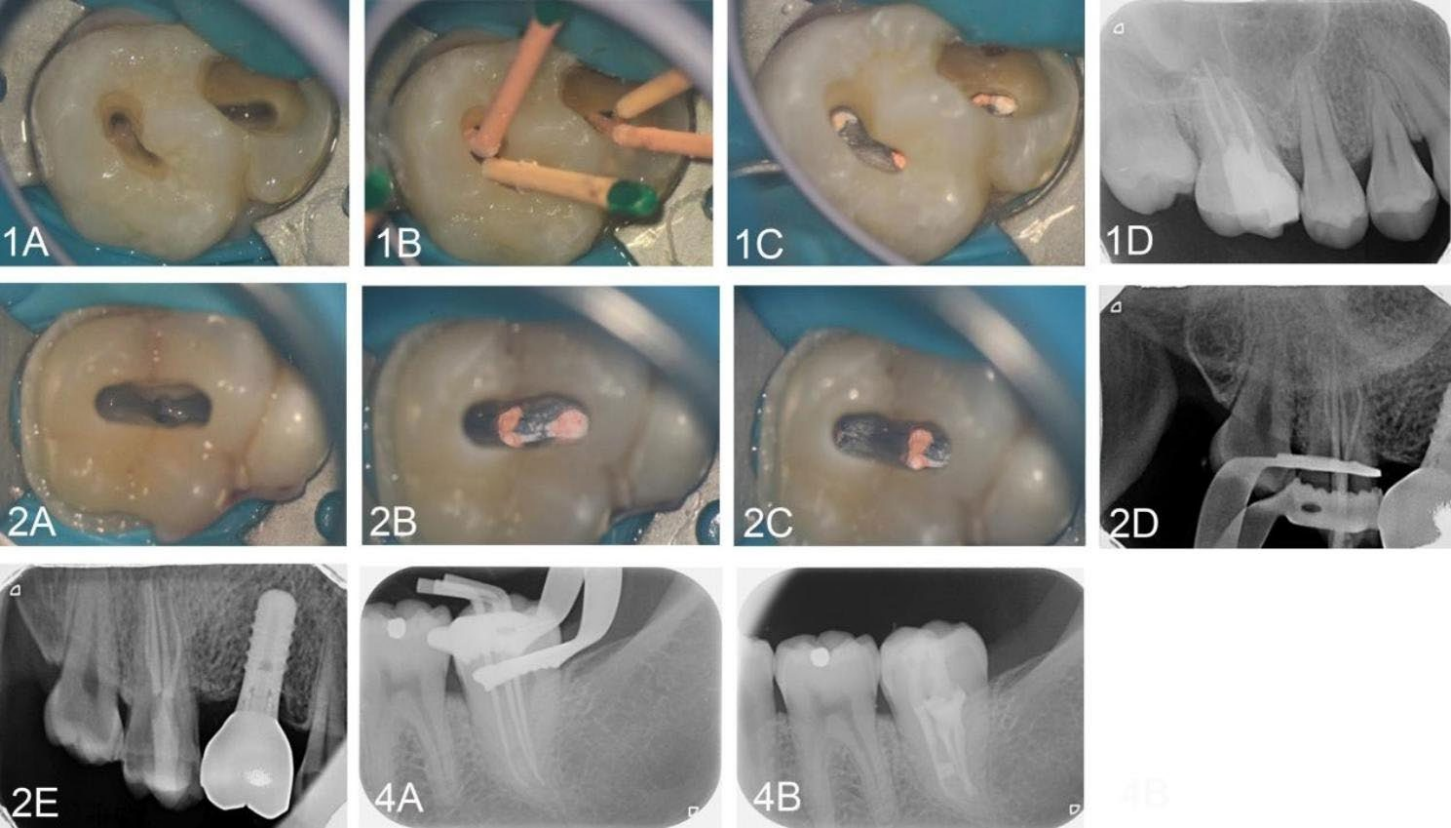
Representative intraoral photos of the diseased teeth during root canal therapy and radiograph of the teeth immediately on completion of endodontic treatment

At the second appointment, 2 weeks later, the tooth was asymptomatic. Complete canal obturation was achieved through the single cone technique with IROOT SP (Innovative BioCreamix Inc, Vancouver, BC, Canada), and the access cavity was then sealed with flow and nanoresin (3 M Dental Products, MN, United States). The final radiograph revealed a dense, well-condensed root filling in all canals. Representative intraoral photographs of the diseased tooth during root canal therapy and radiographs immediately on completion of the treatment were shown in Fig. 6.

For the first case; 1 A: Occlusal view of the endodontic access following the Cleaning and shaping; 1B: Occlusal view of the endodontic access after placement of the gutta-percha point; 1 C: Orifices of the four root canals of tooth #16 with obturation under the microscope; 1D: Post-obturation radiograph.

For the second case; 2 A: Occlusal view of the endodontic access following the Cleaning and shaping; 2 A-2B: Orifices of the four root canals of tooth #17 with obturation under the microscope; 2D: Working length determination radiograph; 2E: Immediate postoperative radiograph.

For the fourth case; 4 A: Working length determination radiograph; 4B: Immediate postoperative radiograph.

## Discussion and conclusions

Geminated and fused teeth are common abnormalities in tooth development that present challenges in differentiating between them and treating their complex root canal systems [7]. Preoperative correct diagnosis is crucial for determining appropriate treatment plans to enhance the efficacy of root canal treatment [8]. Geminated teeth typically have completely or partially separated crowns, while they share the same root and root canal system. Fused teeth result from the fusion of two normal tooth germs, and the pulp chamber and root canal may exhibit various connections or separations depending on the developmental stage at the time of fusion. Nonetheless, the dentin remains connected in fused teeth [1]. Fused teeth can occur between two normal teeth or between a normal tooth and a supernumerary tooth, making it difficult to distinguish them from geminated teeth [9]. Some scholars suggest diagnosing abnormal teeth as “geminated teeth” if the total number of teeth in the dental arch is the same as in a normal arch; otherwise, they are labeled “fused teeth“ [9]. However, this diagnostic approach may not apply in cases where fusion involves natural and supernumerary teeth, as highlighted in cases 2, 3, 4, and 5. Therefore, radiological examination and CBCT imaging are necessary for accurate diagnosis [10]. Nevertheless, many researchers argue that differentiating between specific types of tooth abnormalities may not have significant clinical implications. Identifying dental anomalies and using the term “double teeth” suffices for timely diagnosis and treatment planning in clinical practice [11, 12]. Despite comprehensive clinical and radiographic assessments before surgery, an accurate diagnosis may only become apparent upon postoperative inspection after decay removal [13].

Geminated and fused teeth can lead to various complications, including caries, pulpal periapical disease, periodontal disease, food impaction, abnormal occlusion, tooth fracture, and crowded dentition. Due to the deep grooves formed by the union of these teeth, bacterial plaque tends to accumulate, especially when the grooves extend below the gum line. This increases the risk of caries, periodontal disease, and may necessitate root canal treatment for diseased teeth [4, 14, 15].

Clinicians faced with double tooth cases should conduct a thorough clinical examination, assessing tooth lesions, pulp conditions, and using imaging analysis to determine causes and sources of periapical infections. Based on this evaluation, they can develop appropriate clinical treatment strategies, considering whether the root canal systems of the double tooth are connected. In cases of complete pulp separation, the vitality of the supernumerary tooth and the fused tooth should be assessed individually. Root canal treatment can be performed solely on the diseased portion, preserving the healthy pulp [16]. For example, Ghoddusi reported a case of fusion between the mandibular second molar and a supernumerary tooth. Since the mandibular second molar exhibited normal responses to thermal, electrical, and percussion stimuli, they performed root canal treatment exclusively on the supernumerary tooth while preserving pulp vitality in the second molar [14]. Mei et al. reported a case of fused maxillary second molar with two paramolars and periodontitis [17]. The infection originated from the isthmus between the maxillary second molar and the two paramolars, so they treated the isthmus using an endodontic approach without damaging the original tooth structure, thereby preserving vital pulp. Most fused teeth feature connected root canal systems [18]. Therefore, permanent pulp damage in one tooth may spread to the other, necessitating root canal treatment for both teeth. In summary, a comprehensive analysis and multidisciplinary approach are necessary for developing clinical treatment strategies for double teeth. It involves assessing pulp condition, shape, position, eruption space, and considering overall oral health factors such as periodontal health, occlusal relationship, and dental arch space. Through effective communication with the patient, a comprehensive analysis can guide the development of the most appropriate treatment plan.

Double teeth are more prone to caries and can progress to periapical periodontitis due to the complexity of their root canal system. Therefore, prevention is crucial to maintain pulp vitality and normal physiological function. Caries risk assessment helps identify risk factors and protective measures to prevent and manage caries. Treatment for double teeth can be approached through the three levels of caries prevention. Before caries occur, patients should be educated about the susceptibility of double teeth and receive instructions on oral hygiene. Risk factors should be controlled, regular oral examinations conducted, and preventive measures such as fluoride use and pit and fissure sealants or preventive resin restoration explained to patients. [19]. In the early stage of caries, early diagnosis and treatment can prevent further decay. In cases of deep caries, timely treatment is required to prevent pulpitis or periapical periodontitis. [19].

The complex root canal system of double teeth requires different treatment approaches. CBCT examinations provide a better understanding of root canal anatomy, [20], facilitating effective cleaning, shaping, and obturation [21]. The use of a dental operating microscope enhances visibility, enabling easier root canal location, negotiation, and thorough removal of infected substances. Microscopic visualization combined with ultrasonic instruments is a safe and effective approach. Ultrasonic tips are useful in conservative access procedures for finding canal openings, accessing narrow and curved canals, and removing attached pulp stones. Thinner and longer tips facilitates work in deeper areas while maintaining clear vision.

During root canal preparation, the movement of the irrigation solution is the only effective way to clean lateral canals and apical ramifications that cannot be mechanically cleaned. Ultrasonics are helpful in cleaning these challenging anatomical features. The combination of an irrigant and ultrasonic vibration generates continuous movement, improving root canal cleaning efficacy [22]. Achieving effective three-dimensional obturation is crucial to inhibit bacterial growth and prevent reinfection. The selection of appropriate filling materials is important in root canal filling. Bioceramic materials possess desirable properties such as apical sealing ability, antimicrobial properties, biocompatibility, chemical stability, radiopacity, and slight expansive tendencies. Their high flowability allows for filling of lateral canals and isthmuses, enhancing the seal of the root canal system [23]. In the mentioned cases, the single-cone technique with an Endosequence Bioceramic Sealer, a filling technique based on root canal sealers, was utilized. The gutta-percha tip acts as a carrier for the sealers, aiding in their distribution in the root canal system, especially in webs and fins. Additionally, the gutta-percha tip allows for post space preparation and potential root canal retreatment.

## Data Availability

All data generated or analysed during this study are included in this published article.

## Abbreviations

CBCT: cone-beam computed tomography
NaOCl: sodium hypochlorite
Ca(OH)_2_: calcium hydroxide
